# An external validation study of the Score for Emergency Risk Prediction (SERP), an interpretable machine learning-based triage score for the emergency department

**DOI:** 10.1038/s41598-022-22233-w

**Published:** 2022-10-19

**Authors:** Jae Yong Yu, Feng Xie, Liu Nan, Sunyoung Yoon, Marcus Eng Hock Ong, Yih Yng Ng, Won Chul Cha

**Affiliations:** 1 Department of Digital Health, Samsung Advanced Institute for Health Science & Technology (SAIHST), Sungkyunkwan University, Seoul, Republic of Korea; 2 Digital and Smart Health Office, Tan Tock Seng Hospital, Singapore, Singapore; 3 Programme in Health Services and Systems Research, Duke-National University of Singapore Medical School, Singapore, Singapore; 4 Health Service Research Centre, Singapore Health Services, Singapore, Singapore; 5 Institute of Data Science, National University of Singapore, Singapore, Singapore; 6 Department of Emergency Medicine, Singapore General Hospital, Singapore, Singapore; 7 Department of Emergency Medicine, Tan Tock Seng Hospital, Singapore, Singapore; 8 Digital Innovation Center, Samsung Medical Center, Seoul, Republic of Korea; 9 Department of Emergency Medicine, Samsung Medical Center, Sungkyunkwan University School of Medicine, 115, Irwon-Ro, Gangnam-Gu, Seoul, 06355 Republic of Korea

**Keywords:** Medical research, Risk factors

## Abstract

Emergency departments (EDs) are experiencing complex demands. An ED triage tool, the Score for Emergency Risk Prediction (SERP), was previously developed using an interpretable machine learning framework. It achieved a good performance in the Singapore population. We aimed to externally validate the SERP in a Korean cohort for all ED patients and compare its performance with Korean triage acuity scale (KTAS). This retrospective cohort study included all adult ED patients of Samsung Medical Center from 2016 to 2020. The outcomes were 30-day and in-hospital mortality after the patients’ ED visit. We used the area under the receiver operating characteristic curve (AUROC) to assess the performance of the SERP and other conventional scores, including KTAS. The study population included 285,523 ED visits, of which 53,541 were after the COVID-19 outbreak (2020). The whole cohort, in-hospital, and 30 days mortality rates were 1.60%, and 3.80%. The SERP achieved an AUROC of 0.821 and 0.803, outperforming KTAS of 0.679 and 0.729 for in-hospital and 30-day mortality, respectively. SERP was superior to other scores for in-hospital and 30-day mortality prediction in an external validation cohort. SERP is a generic, intuitive, and effective triage tool to stratify general patients who present to the emergency department.

## Introduction

Emergency department (ED) triage is a critical process for emergency patients who need appropriate treatment and for hospitals that need optimal resource allocation^1,2^. During a pandemic, ED triage is much needed to distinguish patients with high acuity, as there was an increase in the number of cases presenting to ED with higher acuity after COVID-19^3^.

Several early warning-scoring systems, such as the National Early Warning System (NEWS) or the Modified Early Warning System (MEWS), have been established to identify the risk of catastrophic deterioration and inpatient deaths^4^. The Canadian Emergency Department Triage and Acuity Scale (CTAS) is a well-recognized and validated triage system that prioritizes patient care by the severity of illness^5^.

Based on the CTAS, the Korean Triage and Acuity Scale (KTAS) was developed to assess the patient’s severity in Korea^6^. Despite its potential, there were some problems, such as dependence on subjective medical staff assessment during ED triage^1,7,8^.

Several digital machine learning-based triage systems have been proposed for ED triage^7,9,10^. However, the black box property of machine learning makes it hard to interpret and implement in real-world situations. Few studies focus on interpretation to solve the black box problem^11–13^.

Interpretable AI includes reasoning processes that can help make AI predictions understandable for triage in ED^14^. Xie et al. developed the Score for Emergency Risk Prediction (SERP) based on the Singapore population^12^. It used the AutoScore framework to generate and interpret the score^13^. However, this was a single-center study, and external validation will be critical for generalization. This study aims to validate the SERP score derived from the Singapore population on the Korean population and compare the prediction result to that of conventional scores for various perspectives.

## Results

**Figure 1:**
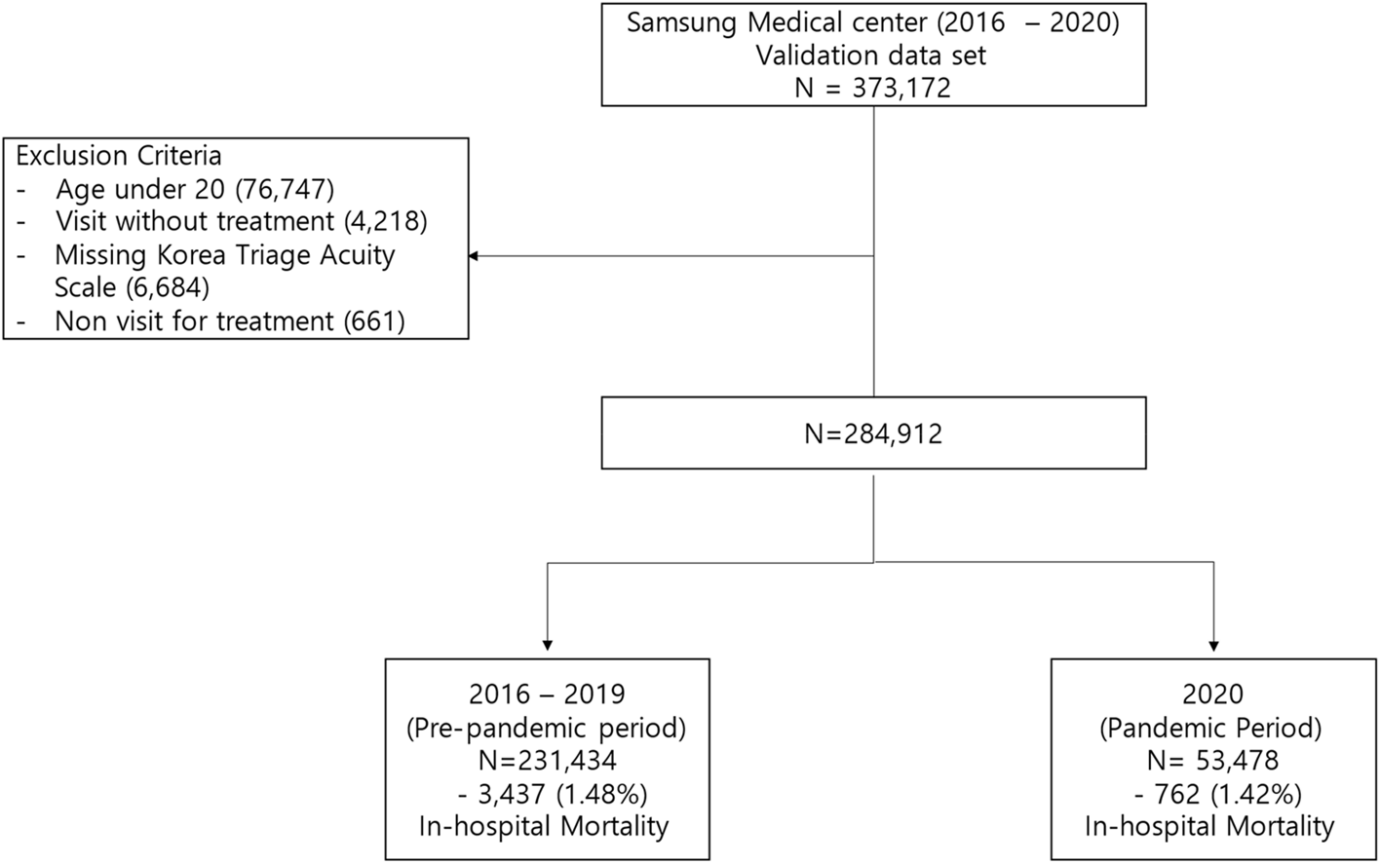
Flow chart of the study population.

As shown in Fig. 1, during the study period from 2016 to 2020 in SMC, 373,172 patients visited the ED. Among them, 87,649 patients were excluded, and 285,523 patients were included in the final analysis (Fig. 1). The mortality rate of the whole cohort was 1.60% for in-hospital death and 3.80% for death at 30 days.

The distribution of ED patients’ demographics is shown in Table 1. The pre-pandemic period cohort included 232,982 ED visits (mean [SD] patient age, 59.9 [17.1] years; 119,681 [51.6%] female). Whereas the pandemic period cohort included 53,541 ED visits (mean [SD] patient age, 56.1 [17.4] years; 27,114 [50.6%] female).

**Table 1:** Baseline characteristics of the validation population.

	Total (n = 263,539)	2016–2019 (Pre-pandemic period) (n = 216,780)	2020 (Pandemic period) (n = 46,759)	*p*-value^a^
Age, mean (SD) (years)	56.2 ± 17.1	56.1 ± 17.1	56.9 ± 17.2	< 0.001
**Sex**				< 0.001
Male	128,499 (48.8%)	105,328 (48.6%)	23,171 (49.6%)	
Female	135,040 (51.2%)	111,452 (51.4%)	23,588 (50.4%)	
**Korean Triage Acuity Scale**				< 0.001
1 (most severe)	1740 (0.7%)	1637 (0.8%)	103 (0.2%)	
2	18,477 (70.%)	15,715 (72.%)	2762 (5.9%)	
3	117,169 (44.5%)	96,446 (44.5%)	20,723 (44.3%)	
4	109,150 (41.4%)	88,796 (41.0%)	20,354 (43.5%)	
5 (less severe)	17,003 (6.5%)	14,186 (6.5%)	2817 (6.0%)	
**Shift time**				< 0.001
8 a.m. to 4 p.m.	122,218 (46.4%)	99,832 (46.1%)	22,386 (47.9%)	
4 p.m. to midnight	95,260 (36.1%)	79,143 (36.5%)	16,117 (34.5%)	
Midnight to 8 a.m.	46,061 (17.5%)	37,805 (17.4%)	8256 (17.7%)	
**Day of week**				< 0.001
Friday	36,710 (13.9%)	30,068 (13.9%)	6642 (14.2%)	
Monday	42,421 (16.1%)	35,089 (16.2%)	7332 (15.7%)	
Weekend	75,638 (28.7%)	62,702 (28.9%)	12,936 (27.7%)	
Midweek	108,770 (41.3%)	88,921 (41.0%)	19,849 (42.4%)	
**Vital signs, mean (SD)**				
Pulse (/min)	88.8 ± 19.5	88.5 ± 19.5	90.1 ± 19.6	< 0.001
Respiration (/min)	18.5 ± 2.5	18.6 ± 2.4	18.1 ± 2.7	< 0.001
SpO_2_ (%)	97.5 ± 3.2	97.5 ± 3.2	97.6 ± 3.2	< 0.001
**Blood pressure (mmHg)**				
Diastolic	131.0 ± 24.9	130.3 ± 24.9	134.1 ± 24.6	< 0.001
Systolic	78.2 ± 15.2	77.5 ± 15.1	81.5 ± 15.3	< 0.001
**Comorbidities**^**b**^				
Myocardial infarction	3,924 (1.5%)	3,275 (1.5%)	649 (1.4%)	0.049
Congestive heart failure	13,805 (5.2%)	11,047 (5.1%)	2,758 (5.9%)	< 0.001
Peripheral vascular disease	6,380 (2.4%)	5,174 (2.4%)	1,206 (2.6%)	0.015
Stroke	24,215 (9.2%)	19,681 (9.1%)	4,534 (9.7%)	< 0.001
Dementia	9,169 (3.5%)	7,564 (3.5%)	1,605 (3.4%)	0.553
Chronic pulmonary disease	19,122 (7.3%)	15,685 (7.2%)	3,437 (7.4%)	0.390
Rheumatoid disease	3,571 (1.4%)	2,925 (1.3%)	646 (1.4%)	0.599
Peptic ulcer disease	14,997 (5.7%)	11,995 (5.5%)	3,002 (6.4%)	< 0.001
**Diabetes**				
Diabetes without chronic complications	28,969 (11.0%)	23,437 (10.8%)	5,532 (11.8%)	0.001
Diabetes with complications	11,201 (4.3%)	9007 (4.2%)	2194 (4.7%)	< 0.001
Hemiplegia or paraplegia	2052 (0.8%)	1593 (0.7%)	459 (1.0%)	< 0.001
Kidney disease	15,625 (5.9%)	12,486 (5.8%)	3139 (6.7%)	< 0.001
**Cancer**				
Local tumor, leukemia, and lymphoma	92,076 (34.9%)	74,592 (34.4%)	17,484 (37.4%)	< 0.001
Metastatic solid tumor	16,903 (6.4%)	13,553 (6.3%)	3350 (7.2%)	< 0.001
**Liver disease**				
Mild liver disease	23,107 (8.8%)	18,704 (8.6%)	4403 (9.4%)	< 0.001
Severe liver disease	3930 (1.5%)	3085 (1.4%)	845 (1.8%)	< 0.001
**Healthcare use, mean (SD)**				
Emergency admissions in the past year	0.3 ± 0.8	0.3 ± 0.8	0.3 ± 0.8	< 0.001
Operations in the past year	0.2 ± 0.6	0.2 ± 0.6	0.2 ± 0.7	< 0.001
ICU admissions in the past year	0.2 ± 0.6	0.2 ± 0.5	0.2 ± 0.6	< 0.001
**Mortality-related outcomes**				
7 days	3289 (1.2%)	2850 (1.3%)	439 (0.9%)	< 0.001
14 days	5474 (2.1%)	4770 (2.2%)	704 (1.5%)	< 0.001
Inpatient	4150 (1.6%)	3415 (1.6%)	735 (1.6%)	0.973
30 days	9921 (3.8%)	8758 (4.0%)	1163 (2.5%)	< 0.001

^a^p-values were calculated using the t-test for continuous and the chi-square test for categorical variables to compare the 2016–2019 and 2020 cohorts.

^b^Comorbidities were calculated for the consideration of the previous 5 years from the ER visit for each patient, and healthcare use was calculated using the previous 1 year.

There were differences between the pre-pandemic and pandemic periods, especially in vital signs and mortality prevalence. Systolic blood pressure and Diastolic Blood Pressure during the pandemic (mean [SD] 130.3 [24.9] and 77.5 [15.1]) were higher than those during the pre-pandemic period (134.1 [24.6] and 81.5 [15.3]). The 30-day mortality was 4.0% during the pre-pandemic period and 2.5% during the pandemic. Regarding the comorbidities, cancer, diabetes, and stroke were the most common diseases. Moreover, patient severity at scene was quite different, the pandemic period saw higher severity patients (1637 (0.8%) vs. 103 (0.2%) (pre-pandemic) for KTAS1, 15,715 (7.2%) and 2762 (5.9%) (pre-pandemic) for KTAS2.

The SERP-30d achieved better performance than KTAS for in-hospital and 30-day mortality prediction, with an AUC of 0.813 (95% CI 0.809–0.817) and 0.795 (95% CI 0.789–0.801), respectively (Table 2). In contrast, KTAS achieved an AUC of 0.717 (0.712–0.722) and 0.741 (0.733–0.749) which results in more than 40% improvement.

**Table 2:** Comparison of AUROC by different scores and outcomes.

AUROC (95% CI)	In-hospital mortality	30-day mortality
SERP-30d	0.813 (0.809–0.817)	0.795 (0.789–0.801)
SERP-7d	0.752 (0.747–0.756)	0.766 (0.759–0.773)
SERP-2d	0.756 (0.751–0.761)	0.782 (0.775–0.789)
KTAS	0.717 (0.712–0.722)	0.741 (0.733–0.749)
CART	0.730 (0.724–0.735)	0.753 (0.745–0.761)
MEWS	0.764 (0.759–0.769)	0.797 (0.790–0.805)
NEWS	0.617 (0.611–0.622)	0.643 (0.634–0.651)
RAPS	0.688 (0.683–0.693)	0.702 (0.695–0.710)
REMS	0.675 (0.670–0.680)	0.722 (0.715–0.728)

*AUROC* Area under Receiver Operating Characteristic, *CI* Confidence Interval, *SERP-nd* Score for Emergency Risk Prediction for predicting n day mortality from admission day in original paper, *KTAS* Korea Triage Acuity Scale, *CART* Cardiac Arrest Risk Triage, *MEWS* Modified Early Warning Score, *NEWS* National Early Warning Score, *RAPS* Rapid Acute Physiology Score, *REMS* Rapid Emergency Medicine Score.

The SERP-30d score showed good calibration (based on the Kolmogorov Smirnov test for calibration data: P = 0.405). The SERP-30d calibration plot on the validation data set is illustrated in Supplementary Fig. 1. As shown in Supplementary Table 2, the results before and after the pandemic period based on 2020 were very different. All SERP performance after the COVID season was superior to that before the COVID season.

In terms of score accuracy, we compared the performance at the same sensitivity and specificity level from 0.7 to 0.9. As shown in Table 3, the SERP score achieved a higher sensitivity than KTAS at the same specificity level. For example, at the same 0.7 sensitivity, the specificity of SERP was 0.790, whereas KTAS was 0.568. This result shows that SERP can detect more patient with a higher mortality risk than KTAS.

**Table 3:** Comparison of prediction model accuracy with same specificity point.

Type	Specificity cut-off value	Sensitivity (95% CI)	PPV (95% CI)
SERP–In-hospital	0.7	0.792 (0.785–0.796)	0.094 (0.093–0.094)
KTAS	0.7	0.524 (0.518–0.531)	0.064 (0.063–0.065)
SERP–In-hospital	0.8	0.673 (0.668–0.683)	0.116 (0.115–0.118)
KTAS	0.8	0.402 (0.397–0.408)	0.073 (0.072–0.074)
SERP–In-hospital	0.9	0.453 (0.443–0.463)	0.151 (0.148–0.153)
KTAS	0.9	0.276 (0.272–0.289)	0.098 (0.096–0.102)

*KTAS* Korea Triage Acuity Scale, *SERP* Score for Emergency Risk Prediction (in-hospital mortality outcome here), *CI* Confidence Interval *PPV* Positive predictive value.

**Figure 2:**
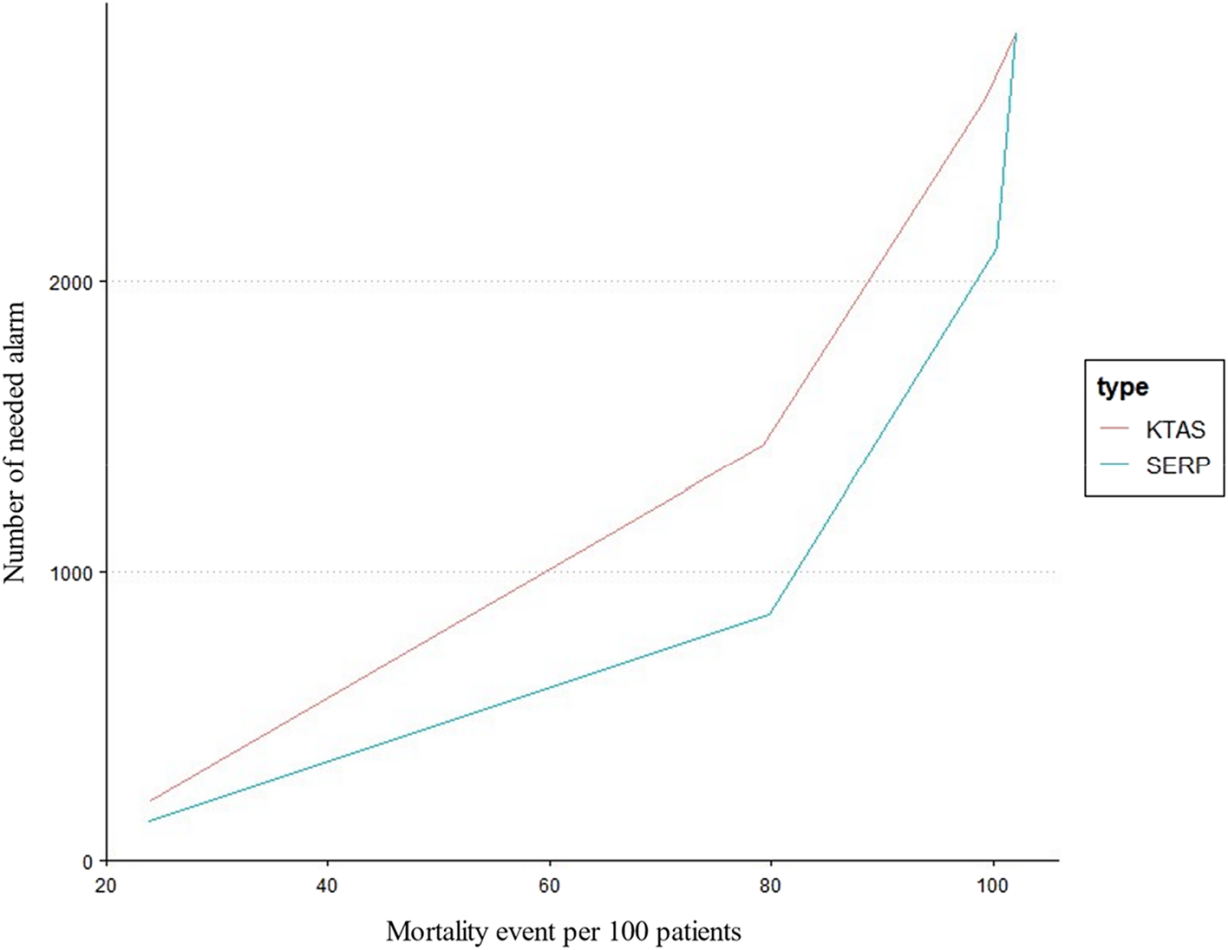
Comparison of the number of needed alarms at the same sensitivity point for predicting mortality between KTAS (Korean Triage Acuity Scale) and SERP (Score for Emergency Risk Prediction).

Regarding the alarm fatigue problem, we compared the performance between scores at the same mortality event occurrence. As shown in Fig. 2, KTAS results in more alarms for the same event than SERP. For example, for 9937 and 7925 events, KTAS raised 263,172 and 143,382 alarms, respectively, whereas the SERP score resulted in only 211,848 and 85,134, a decrease of 19% and 40% of alarms, respectively.

## Discussion

We validated the SERP score to predict mortality in the ED using SMC data. The results of SERP in our main two aspects (performance and alarm fatigue) were better than conventional ED triage scores. Also, SERP resulted in fewer false alarms for the same event occurrence. Excessive false alarms can reduce productivity and result in alarm fatigue, putting critical patients at risk^15^.

Previous studies on machine learning usually focus on accuracy^7,9,10^. However, only a few studies have demonstrated interpretability for easy use of the model. One of the critical points is the importance of real-world application. In the complex and busy ED environment, it is necessary to make the model light and interpretable. The other strength of SERP is that it requires few features for development. The features in the SERP are routinely collected during triage—so implementing the SERP score in the ED is not a big challenge.

There is growing consensus among researchers related to efforts for the real-world application of AI in healthcare and practical issues regarding the implementation of AI into existing clinical workflows^16,17^. Brajer et al. suggested a machine-learning model fact sheet reporting for end-users^18^. Visualization-based efforts such as population, patient, and temporal level feature importance, or nomograms, could be adopted^19–22^. Like the Transparent reporting of a multivariable prediction model for individual prognosis or diagnosis (TRIPOD) or The Strengthening the Reporting of Observational Studies in Epidemiology (STROBE) guidelines for reporting machine learning results^23,24^, there should be a guideline for the standardization of user interfaces (UIs) and a format for clinical decision support for end-users, including clinicians and patients^25,26^. In terms of data-sharing, privacy, and interoperability across multiple platforms, hospital policies and national laws are also important. Lack of standardization, black box transparency, proper evaluation, and problems with patient safety are the other major key issues for AI implementation^16,17^.

The characteristics of the patient populations could be quite different in different hospitals and countries. Although the SERP validation performance was good for long-term outcomes, conventional indexes such as NEWS, MEWS, and KTAS were equivalent for short-term outcomes. There may be a role for customization of a new SERP score for Korea. We recognized that as the mortality timeframe increased from 2 to 30 days, the performance worsened in the conventional indexes but improved in the ML-based score.

The subgroup analysis showed a difference in the performance between the pandemic and pre-pandemic periods. This could be due to the different patient mix during the pandemic^27,28^. We also identified differences in feature importance between the pandemic and pre-pandemic periods. During the COVID season, the top three important features were related to vital signs, whereas age was the second most important variable during the pre-pandemic period. Finally, the rate of admission and transfer were higher during the pandemic, even though patient illnesses were less severe based on KTAS.

There are some limitations to this study. First, it is a retrospective study and needs to be further evaluated prospectively, although the strengths of this validation are the multi-center and multi-nation nature of this evaluation. Second, we only considered Korean SMC data, which may not represent all Koreans. In the future, we intend to conduct the same validation with more hospitals in Korea or the National Emergency Department Information System (NEDIS), which is a nationwide registry of ED data^29^. As the variable used for SERP score is not complicated, we can consider international validation of the score, applying to other nationwide registry ED using Common Data Model or Pan Asia Trauma Outcome Study.

In this study, we validated the SERP score with Korean data. Its performance was better than the conventional indexes in terms of accuracy and false alarms.

## Methods

### Study setting

This was a retrospective validation study of the SERP score using data from the Samsung Medical Center (SMC) in Korea. SMC is a tertiary hospital located in a metropolitan city in Korea. The hospital has approximately 2000 inpatient beds. More than 80,000 patients visit the ED annually.

The Electronic Health Records (EHR) were obtained from the Clinical Data Warehouse at SMC. This study was approved by the Samsung Medical Center Institutional Review Board (2022-05-083-001), and a waiver of consent was granted for EHR data collection and analysis because of the retrospective and de-identified nature of the data.

All methods were performed in accordance with the relevant guidelines and regulations^24^.

### Population

The population for the validation cohort was ED visits from 2016 to 2020. All patients who visited the ED from January 2016 to December 2020 were initially included. We excluded patients who were under the age of 20 years, did not come for emergency treatment, left without being seen by a clinician, had missing triage data, or were dead on arrival (DOA) (see Fig. 1)^15^. To assess the impact of the COVID-19 pandemic, we defined two non-overlapping cohorts based on “pre” and “post” pandemic periods.

### SERP score

Three SERP scores were validated using the primary outcomes of 30-day and in-hospital mortality from the ED visits. Each score was developed using the AutoScore framework, which is an automatic and interpretable score generator for risk prediction using machine learning and logistic regression^12,13^.

For outlier data, we assumed that extreme ranges of vital sign data were input errors and designated them as “missing” based on clinical knowledge. For example, any vital signs value under 0, heart rate above 300/min, respiration rate above 50/min, systolic blood pressure above 300 mm Hg, diastolic blood pressure above 180 mm Hg, or oxygen saturation as measured by pulse oximetry above 100% were treated as a missing value and imputed with the median value from a training cohort. Missing rates of each variable are presented in the “Supplemental Tables S1”.

### Statistical analysis

The data were analyzed using R software, version 3.5.3 (R Foundation for Statistical Computing).

For the descriptive summaries of baseline characteristics of the study population, frequency (percentages) for categorical variables and mean (SD) for continuous variables were reported.

### Performance evaluation

We compared the validation performance of SERP with conventional indexes such as NEWS, MEWS, and KTAS, in terms of two main aspects^4^. First, how accurately can the SERP score predict the outcome compared to a conventional index? The predictive power of validation was measured using the AUC in the receiver operating characteristic (ROC) curve. Other metrics such as sensitivity, specificity, and positive predictive value, were calculated under a certain threshold from 0.7 to 0.9 for the comparison. We also identified the calibration plot for the agreement between predictions and the observed outcome^30^. Second, can SERP reduce the false alarm rate more than the conventional index? The alarm rate is important for the validation of SERP because false alarms can result in alarm fatigue^31^. Alarm fatigue can make medical staff tired and cause critical alerts to be missed. Finally, it could affect patient safety and quality of care in the clinical environment. Therefore, an ideal SERP should have high sensitivity and a low false alarm rate. We compared the frequency of alarming events with the KTAS.

## Supplementary Information

Supplementary Tables.

## Data Availability

Data was available in study site clinical data warehouse. The datasets generated and analyzed during the current study are not publicly available due dataset includes although is de-identified, part of patient information, but are available from the corresponding author on reasonable request.

## Abbreviations

ED: Emergency department
SERP: Score for emergency risk prediction
SMC: Samsung Medical Center
EHR: Electronic health record
DOA: Dead on arrival
KTAS: Korean triage acuity scale
CART: Cardiac arrest risk triage
NEWS: National early warning system
MEWS: Modified early warning system
RAPS: Rapid Acute Physiology Score
REMS: Rapid Emergency Medicine Score
CTAS: Canadian Emergency Department Triage and Acuity Scale
AUROC: Area under the receiver operating characteristic curve
SD: Standard deviation
CI: Confidence interval
NEDIS: National Emergency Department Information System of Korea
TRIPOD: Transparent reporting of a multivariable prediction model for individual prognosis or diagnosis
STROBE: The Strengthening the Reporting of Observational Studies in Epidemiology
UI: User interfaces
