# Supplementary material for: An external validation study of the Score for Emergency Risk Prediction (SERP), an interpretable machine learning-based triage score for the emergency department
Source: Sci Rep. 2022 Oct 19;12:17466. doi: 10.1038/s41598-022-22233-w (PMC9580414; doi:10.1038/s41598-022-22233-w)
Supplement: Supplementary file 1 — Supplementary Tables. [file 41598_2022_22233_MOESM1_ESM.docx]

E Table 1. The numbers (percentage) of the missing variables for the validation cohort.

|  | **n** | **Missing frequency (%)** |
| --- | --- | --- |
| Age | 263,539 | 0 (0.0%) |
| Sex | 263,539 | 0 (0.0%) |
| Shift time | 263,539 | 0 (0.0%) |
| Day of week | 263,539 | 0 (0.0%) |
| Vital signs |  |  |
| Pulse, /min | 263,539 | 3,239 (1.22%) |
| Respiration, /min | 263,539 | 3,585 (1.36%) |
| SpO_2_, % | 263,539 | 7,502 (2.84%) |
| Diastolic | 263,539 | 4,040 (1.53%) |
| Systolic | 263,539 | 4,031 (1.53%) |

*SpO_2_* Oxygen saturation

E Table 2. Comparison of AUROC by different scores and outcomes for normal and pandemic periods.

|  | **Pre-pandemic period** | | **Pandemic period** | |
| --- | --- | --- | --- | --- |
| **AUROC (95% CI)** | **Inhospital** | **30d** | **Inhospital** | **30d** |
| SERP-30d | 0.814 (0.81–0.818) | 0.793 (0.786–0.8) | 0.809 (0.798–0.82) | 0.803 (0.79–0.817) |
| SERP-7d | 0.75 (0.745–0.755) | 0.764 (0.757–0.772) | 0.763 (0.75–0.776) | 0.774 (0.758–0.79) |
| SERP-2d | 0.753 (0.748–0.758) | 0.78 (0.772–0.788) | 0.774 (0.761–0.787) | 0.79 (0.774–0.806) |
| KTAS | 0.716 (0.711–0.721) | 0.74 (0.732–0.749) | 0.736 (0.722–0.75) | 0.745 (0.727–0.763) |
| CART | 0.727 (0.722–0.733) | 0.75 (0.741–0.759) | 0.749 (0.735–0.764) | 0.766 (0.748–0.784) |
| MEWS | 0.76 (0.755–0.765) | 0.796 (0.788–0.804) | 0.784 (0.77–0.799) | 0.802 (0.785–0.82) |
| NEWS | 0.617 (0.611–0.623) | 0.643 (0.634–0.653) | 0.624 (0.608–0.64) | 0.639 (0.619–0.658) |
| RAPS | 0.69 (0.684–0.695) | 0.705 (0.697–0.713) | 0.69 (0.675–0.704) | 0.69 (0.671–0.708) |
| REMS | 0.67 (0.664–0.675) | 0.722 (0.715–0.729) | 0.705 (0.692–0.719) | 0.721 (0.706–0.737) |

*AUROC* Area under Receiver Operating Characteristic, *CI* Confidence Interval, *SERP-nd* Score for Emergency Risk Prediction for predicting n day mortality from admission day in original paper, *KTAS* Korea Triage Acuity Scale, *CART* Cardiac Arrest Risk Triage, *MEWS* Modified Early Warning Score, *NEWS* National Early Warning Score, *RAPS* Rapid Acute Physiology Score, *REMS* Rapid Emergency Medicine Score
